# Towards Digital Manufacturing of Smart Multimaterial Fibers

**DOI:** 10.1186/s11671-019-3031-x

**Published:** 2019-06-18

**Authors:** Camila Faccini de Lima, Louis A. van der Elst, Veda Narayana Koraganji, Mengxin Zheng, Merve Gokce Kurtoglu, Alexander Gumennik

**Affiliations:** 1 Department of Intelligent Systems Engineering, School of Informatics, Computing and Engineering, Indiana University, 700 North Woodlawn Avenue, Bloomington, Indiana 47408 USA; 2 Fibers and Additive Manufacturing Enabled Systems Laboratory, 2425 North Milo B. Sampson Lane, Bloomington, IN 47408 USA

**Keywords:** VLSI, Fiber devices, Multimaterial fibers, Microstructures, 3D printing of glass, Fluid dynamics, Biomimetic scaffolds

## Abstract

Fibers are ubiquitous and usually passive. Optoelectronics realized in a fiber could revolutionize multiple application areas, including biosynthetic and wearable electronics, environmental sensing, and energy harvesting. However, the realization of high-performance electronics in a fiber remains a demanding challenge due to the elusiveness of a material processing strategy that would allow the wrapping of devices made in crystalline semiconductors, such as silicon, into a fiber in an ordered, addressable, and scalable manner. Current fiber-sensor fabrication approaches either are non-scalable or limit the choice of semiconductors to the amorphous ones, such as chalcogenide glasses, inferior to silicon in their electronic performance, resulting in limited bandwidth and sensitivity of such sensors when compared to a standard silicon photodiode. Our group substantiates a universal in-fiber manufacturing of logic circuits and sensory systems analogous to very large-scale integration (VLSI), which enabled the emergence of the modern microprocessor. We develop a versatile hybrid-fabrication methodology that assembles in-fiber material architectures typical to integrated microelectronic devices and systems in silica, silicon, and high-temperature metals. This methodology, dubbed “VLSI for Fibers,” or “VLSI-Fi,” combines 3D printing of preforms, a thermal draw of fibers, and post-draw assembly of fiber-embedded integrated devices by means of material-selective spatially coherent capillary breakup of the fiber cores. We believe that this method will deliver a new class of durable, low cost, pervasive fiber devices, and sensors, enabling integration of fabrics met with human-made objects, such as furniture and apparel, into the Internet of Things (IoT). Furthermore, it will boost innovation in 3D printing, extending the digital manufacturing approach into the nanoelectronics realm.

## Introduction

Although glass-drawn fibers date back to the Roman times, the first functional optical fibers were manufactured in 1792 by the French Chappe brothers for communication purposes [1]. In 1842, Jean-Daniel Colladon, a Swiss physicist, showed that light could be guided internally through a water jet [2]. These two discoveries sprouted decades of optical and material engineering improvements leading to today’s efficient fibers, enabling high-speed telecommunication and data storage across kilometers of distance around the world, such as the 25,000-km-long trans-Pacific undersea cable (TPC) completed in 1996 [3, 4]. Moreover, fiber optic sensors (FOS) are used for a wide variety of biomedical, oil and gas, marine, architectural, chemical, and aerospace applications [5, 6].

The control and improved performance of light propagation using photonic crystals fibers (PCF) developed in 1996 by Philip Russel opened the doors for research in more complex fiber internal functional structures [7–9]. New microstructures also welcomed the integration of material diversity in their constitution [10, 11] to design smart fibers for electronics [12], optoelectronics [11], in-fiber synthesis [13], microfluidics [14], microelectromechanical systems [15, 16], and biosynthetic interfacing [17]. Smart fibers differ from traditional fibers by including a non-traditional function beyond optical communication and the typical usage of fibers in commercial fabrics. Smart fibers can be used for esthetics in electronic textiles by controlling the colored appearance of the fibers due to optical interference in their microstructure [18] or for enhancing performance, for example in the case of conventional optical fiber guides and dielectric mirror lining allowing light guidance through air [19, 20].

To make a fiber functional, it should be comprised of materials with varying electronic properties, its architecture should be specifically designed to perform a given function, and its internal features should be reduced at the nanoscale, orders of magnitude smaller than the core of current telecommunication fibers. Fibers are typically created by optical fiber fabrication methods, that is, being thermally drawn from macroscopic cylindrical or cuboid rods called “preforms”. The fabrication process begins with a selection of appropriate core and cladding materials, such as metals, insulators, and semiconductors. For example, the first fiber including metal-insulator-semiconductor structures was developed in 2004 for photodetection [21]. Materials for the preform are selected such that their viscosities, *μ*, are comparable at draw temperature, falling roughly in the window of 4 < log(*μ*)[poise] < 6. This is required to prevent shear flows and capillary instabilities that otherwise distort the fiber device geometry. Materials for which this requirement is unattainable, such as metals or crystalline semiconductors that are very thin in their liquid form during the fiber draw, must be confined to channels with a low aspect ratio, with geometry close to equilibrium.

**Fig. 1:**
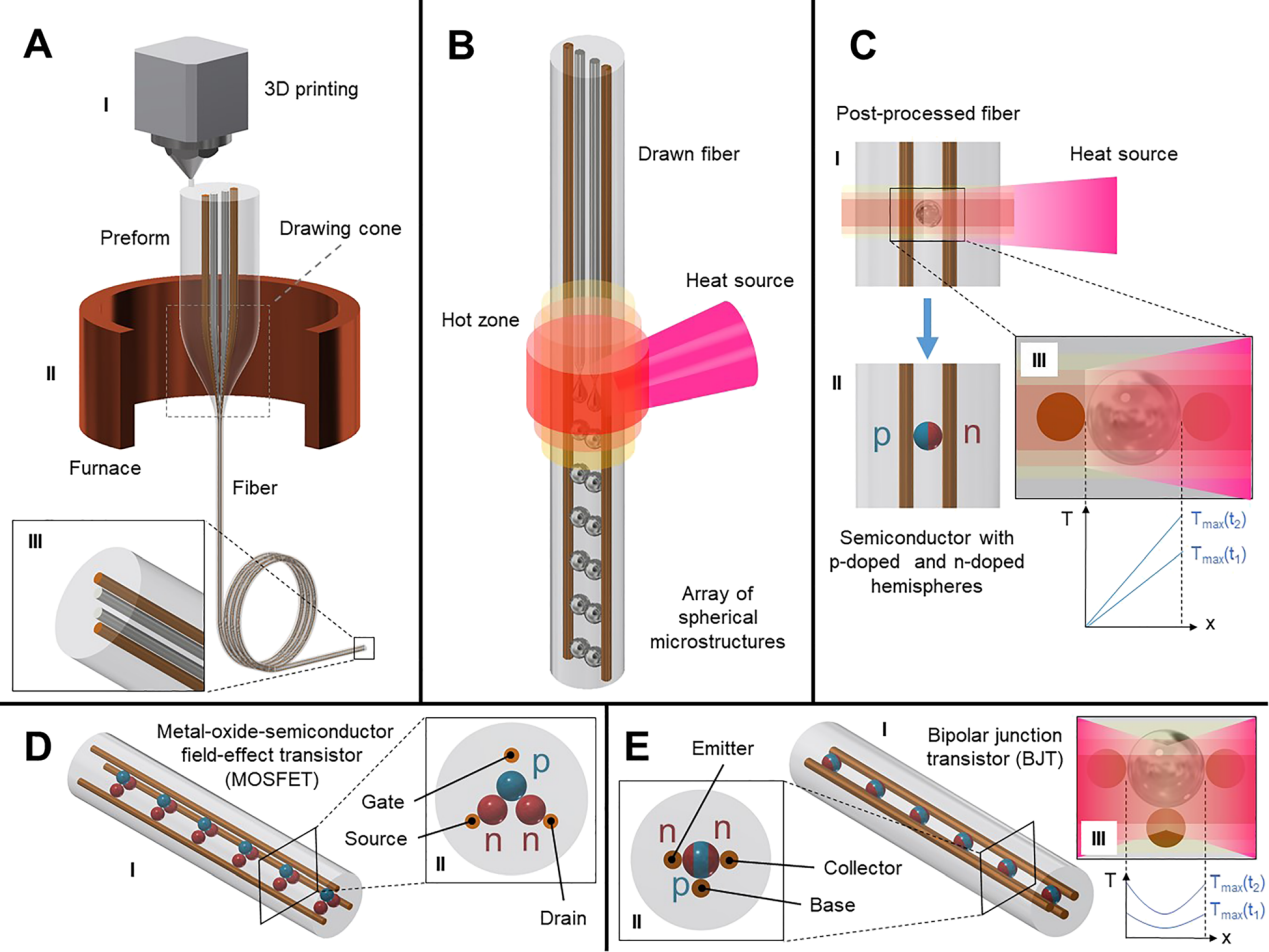
VLSI-Fi: Conceptual schematics of the VLSI-Fi technique representing the “2D + 1D + 0D” approach**. a** The 3D-printed preform **a** (I) is thermally drawn **a** (II) into a long, thin fiber that preserves the cross-sectional geometry of the preform (2D). **b** Axial patterning of the fiber via spatially coherent, material-selective capillary breakup (+1D), resulting in the assembly of initially continuous, separate cores into arrays of discrete devices contacted in parallel. **c** Segregation-driven control of doping in post-breakup semiconducting particles, allowing control of an individual device’s internal architecture **c** (II) via thermal gradient **c** (III). **d** (I) Schematic illustration of Metal-oxide-semiconductor field-effect transistor (MOSFET) through VLSI-Fi, where the p-type and n-type semiconductors are shown in blue and red, respectively. The golden continuous rods embedded in a silica fiber act as gate, source, and drain. The resulting fiber cross section is shown in **d** (II). Similarly, **e** (I) shows a schematic picture of a bipolar junction transistor (BJT) realized by VLSI-Fi, achieved with impinging heat sources from both the emitter and collector sides. The fiber cross section **e** (II) shows the emitter, collector, and base of the BJT (continuous rods embedded in the fiber), with the p-type and n-type semiconductors of the n-p-n junction shown in blue and red, respectively

The preform, which is essentially a scaled-up version of the fiber, can be built using a variety of techniques such as rolling sheets of material like a rug, stacking milled parts like a puzzle, or 3D printing, as shown in Fig. 1a (I) and discussed in this review, and then consolidated by a vacuum sintering. The preform is then heated in a furnace and elongated into a fiber like a caramel or taffy (see drawing cone in Fig. 1a (II)), while preserving its cross-sectional arrangement based on a construct’s given thermomechanical properties such as viscosities, interface energies, mutual adhesion, and differential thermal expansion (Fig. 1a (III)) [22]. This process, for the case of a 3D printed preform, is schematically illustrated in Fig. 1a. A typical draw can yield kilometers of fiber with very fine nanoscale cross-sectional diameters around 5 nm [23, 24].

In a post drawing step, playing on capillary instabilities, the fiber can be reliquefied by heating to allow for the breakup of the cores in a spatially coherent material-selective manner, enabling an axial control over the fiber-embedded structures [25–27] as illustrated in Fig. 1b. Alternative techniques for patterning the cores axially include UV-exposure through photomasks in photopolymeric cores, resulting in non-trivially-shaped microparticles [28]. Other hybrid-functionalization techniques include coating the fiber surfaces with functional materials [10, 29] and confinement of a fiber cladding by a draw [12] to an array of optoelectronic devices fabricated by standard complementary metal-oxide-semiconductor (CMOS) manufacturing.

Fibers can be weaved into fabrics or nets to attain collective functionality that surpasses that of an individual fiber [30]. Designed through biomimicry, fibers can be shaped according to nature’s useful features to enhance the fabric’s functionality, such as hydrophobicity [31]. The fibers can also be conceived as synthesis platforms for inexpensive material production such as the conversion of aluminum cores into silicon in silica-cladded fibers [13] or thermally induced fabrication of porous structures by phase separation [32]. Fibers can also serve remote and distributed signal detection, such as environmental chemical sensing of hazardous volatiles [29]. The flexibility in fiber design is such that multiple functional modalities can be integrated in one fiber for complex applications such as deep spinocortical stimulation and monitoring in mice for neurodegenerative diseases research [33]. These examples show some of the variety of domains in which smart fibers are the natural solution.

### Motivation

Even though fibers are universally ubiquitous, the integration of high-performance microelectronic systems within a thin-fiber remains a major challenge [11, 12]. Different approaches addressing this challenge have been proposed, with efforts mostly focused on low-temperature materials. For example, high-pressure chemical vapor deposition (HPCVD) has been used to integrate compound semiconductors in microstructured optical fibers (MOFs) [34], as well as to create flexible silicon p-i-n junction fibers [35]. Alternatively, a CO_2_ laser was used for the recrystallization of SiGe core in silica fibers to engineer their electronic properties [36]. Such approaches either result in devices with limited electronic bandwidth, as is the case with chalcogenide materials, which are intrinsically amorphous or are inherently non-scalable.

As such, the Fibers and Additive Manufacturing Enabled Systems Laboratory (FAMES Lab) has developed and currently implements a technique for controlling the 3D architecture of fibers, which is described in this review. Since the intricacy of the final fiber correlates to the complexity of the preform cross section, free-form fabrication of preforms enables fiber device functionalities unattainable otherwise. Moreover, 3D printing is widely accessible, making it a cost-effective and user-friendly technological alternative to traditionally employed methods, allowing for a wide range of materials, from thermoplastics to high-temperature materials, as well as biomaterials [37].

In addition, the FAMES Lab has the ability to process high-temperature materials, alongside the more traditional use of thermoplastics in additive manufacturing, allowing us to take advantage of properties such as the high electron mobility in Si/Ge [38], as well as the future use of lead zirconate titanate (PZT)/BaTiO_3_ composites for piezoelectric applications having large piezoelectric coefficients compared to polymers [39, 40] and higher acoustic bandwidth [41].

With these challenges in mind, we propose optimization solutions using additive manufacturing to achieve faster and more complex preform fabrication, capillary breakup simulations to optimize axial control of the fiber, and combinations of our fibers with tissue engineering. These strategies enable the creation of realistic biomedical platforms with biosensing and biofunctionalizing capabilities for drug and treatment analysis in vitro as one of the promising fiber device applications.

### Concept

In order to realize functional fibers, we draw inspiration from very large-scale integration (VLSI)—a digital design and manufacturing technique that gave rise to the modern microprocessor in the 1970s. This technique uses photolithography and chemical/thermal treatment of exposed areas of the semiconducting substrate wafer to define the features of the integrated circuit in this layer (2D) while the fabrication of a complete circuit progresses by stacking of such individual layers in the direction perpendicular to the wafer surface (+1D). Additionally, the electronic doping of individual components of the circuit (+0D) can be controlled by implantation and thermal activation [42]. Our approach to fibers, dubbed “VLSI for Fibers” or “VLSI-Fi,” is analogous: first, additive manufacturing and thermal draw of a preform define the cross-sectional geometry of the fiber device (2D); second, the resulting fiber can later be axially patterned (+1D), allowing for the assembly of arrays of integrated discrete devices from initially continuous but separated cores; and third, segregation-driven structuring of individual in-fiber-embedded devices (0D) can be performed. Table 1 draws a comparison between the two techniques, highlighting the correspondence between each degree of geometry control made possible by each technique (“2D + 1D + 0D”). The schematics of the described steps are shown in detail in Fig. 1, where examples of possible in-fiber-embedded devices could be realized using the VLSI-Fi technique.

**Table 1:** Comparison table between VLSI and the analogous approach for fibers (VLSI-Fi)

	VLSI	VLSI-Fi	Graphical illustration
2D	Wafer surface by photolithography and chemical and thermal treatment	Fiber cross section by 3D printing of preforms and thermal draw	Fig. 1a
+1D	Layer-by-layer structuring by vertical stacking	Axial patterning by capillary breakup	Fig. 1b
+0D	Doping control by implantation and thermal activation	Segregation-driven structuring by temperature gradient-guided solidi cation	Fig. 1c

### Experimental Section

In this section, we first describe our work on the 3D printing of polycarbonate preforms, followed by recent advances in glass 3D printing using stereolithography. Then, we approach the axially patterning of the fibers through a spatially coherent, material-selective capillary breakup, which allows for the assembly of initially continuous, separate cores into arrays of discrete devices contacted in parallel. Finally, we propose the application of our biointerfacing fibers, combined with tissue engineering to monitor viable tissue growth in vitro. Functions are enabled by using in-fiber microfluidic channels to deliver cells and signaling biochemicals and shape memory alloy wires for movement control, and piezoelectric elements to map the environment by ultrasound waves.

### Draw of 3D-Printed Preforms

As stated previously, conventional preform fabrication techniques, such as thin-film-rolling and stack-and-raw [10], are limited in producing complex geometrical structures, take up a significant amount of time in the fiber draw process, and require skilled labor and expensive equipment. 3D printing addresses these problems with the help of soluble support material and its partly automated and user-friendly process. This allows the printing of very complex geometries with ease in a relatively short period of time.

In order to assess the influence of the printing angle of polycarbonate (Hatchbox 3D) preforms, cylindrical and square-shaped rods were printed using a single head Prusa i3 MK2 FDM-printer. The extruder and the printbed temperatures were set to 235 °C and 105 °C, respectively, and the printer was set to produce 100% infill preforms with a 0.35-mm nozzle. The orientation of the layers in the preform depends on the horizontal orientation of the part with respect to the print bed, and when the preform is introduced into the furnace during the thermal draw, the heat flow is affected by the layer orientation. To test which orientation was best suited for thermal draw, preforms with 0°, 15°, 30°, 45°, and 90° orientations were printed. All angles were measured between the longitudinal axis of the preform and the horizontal axis. The 3D-printed preforms underwent the thermal draw process in a furnace with three temperature zones of 90 °C, 100 °C, and 200 °C. For the cylindrical preforms, the thermal draw was successful at angles 0° and 45°. The 90° preform draws always fail due to layer delamination.

**Fig. 2:**
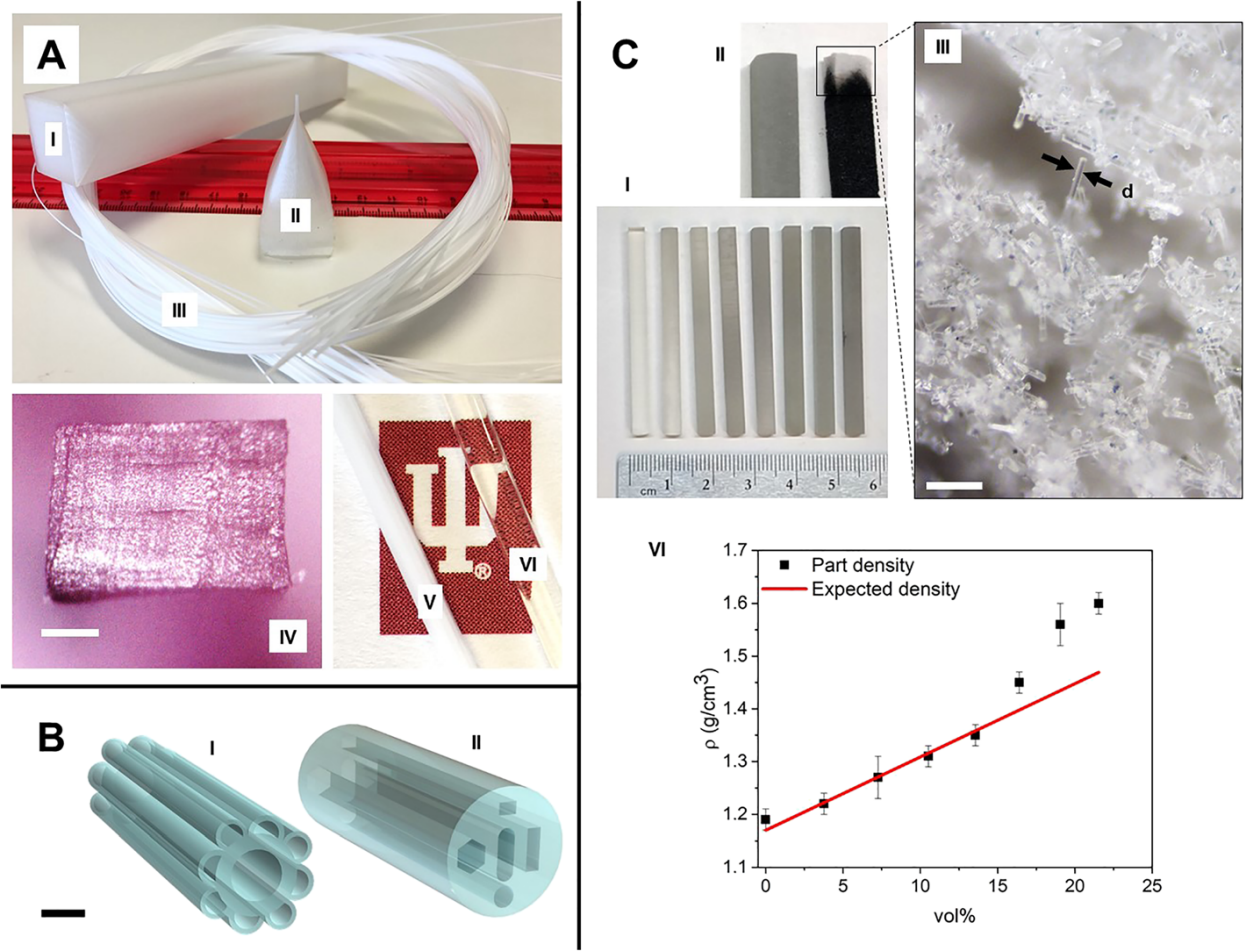
3D printed preforms: **a** (I) Square-base 3D printed polycarbonate preform. **a** (II) Drawing cone. **a** (III) Resulting polycarbonate fiber after thermal draw. **a** (IV) Fiber cross section after the draw process, with no layer delamination. The cross section is rectangular due to non-isotropic porosity of the infill pattern (scale bar 200 μm). **a** (V) Drawn fiber before annealing. **a** (VI) Drawn fiber after annealing with apparent optical transparency. **b** CAD models of Glass preforms, successfully realized in soda-lime glass with high-precision extrusion-based 3D printing. **b** (I) Structure mimics blue tarantula hair. **b** (II) Preform model with non-equilibrium cross-sectional geometry (scale bar 1 cm). **c** (I) Square-shaped glass samples with increasing glass infill (from left to right), printed with SLA technique. **c** (II) Detail of glass sample before baking (left) and during baking (right). In the latter, it is possible to see the black coloration resulting from the carbonized residues of resin, while the tip presents white coloration after these residues are ashed out. **c** (III) Picture under microscope of the ashed-out section (scale bar 200 μm), where the white coloration is a result of the natural color of the compacted milled fibers in an interconnected porous structure. Moreover, the nominal width of the glass fiber, indicated in the image by d, correlates to the expected values of 16 μm (#38 Fiber Glast). **c** (IV) A plot of the printed glass preforms densities (*ρ*) as function of the volume fraction of glass fibers mixed with resin, along with the average density of the print material

We were also able to successfully draw non-equilibrium structures such as a square rod with an improvised fill pattern as shown in Fig. 2a (I), where instead of the rectilinear fill pattern, the infill was also set to follow the perimeters and print in ordered structure. The 0° square-based preform draw was successful, and although it deformed slightly, the fiber was still able to retain the shape of the preform. Fibers with dimensions as low as 40 μm × 60 μm were successfully drawn and are shown in Fig. 2a, having no delamination of layers. Furthermore, all the layers were consolidated properly. An example of a fiber cross section is shown in Fig. 2a (IV), and images of a fiber before and after annealing are shown in Fig. 2a (V) and 2a (VI), respectively, where we see that the annealed fiber achieves optical transparency, which will be characterized in future work. From these experiments, we conclude that the most successful preforms are the ones printed at 0°, whereas 90° always delaminate during the draw process. The consolidation of layers in the thermal drawn fibers improved as the orientation angle of the 3D printed preform decreased.

### 3D Printing of Glass Preforms

Beyond thermoplastics, glasses including fused quartz have significant scientific and engineering applications in optics, communications, and electronics [43]. Structured silica fibers could benefit multiple applications, for example dye-less coloration of fabrics for fashion, photonic crystal fibers for optical-chemical detection, or single-mode fibers for telecommunication and tight focusing of light. These fibers are generally fabricated by a 2-step process: the preform fabrication and the thermal draw of the preforms into a fiber. While the draw process is relatively simple and cheap, the preform fabrication, at this point, requires case-by-case treatment, and for each specific preform configuration, a separate technology must be developed and applied.

Figure 2b shows soda-lime glass preform computer-aided design (CAD) models that were realized successfully with high precision in extrusion-based printing technology similar to the products described by the company Micron3DP [44]. This material has promising optical properties especially in long wavelengths such as IR [45], making it an interesting candidate for the fabrication of fibers with novel optical functionalities. The preform model shown in Fig. 2b (I) mimics the structure of the blue tarantula hair, similar to the structure presented in [46], and in Fig. 2b (II), the preform cross section contains non-equilibrium geometries and is thus prone to reshaping due to surface tension minimization. These models were designed and realized as a verification of the possibility of achieving complex, non-equilibrium preform cross sections.

Since the cladding structure provides mechanical integrity to the fiber during the draw process—being composed of the most viscous material—our efforts so far have been focused on this component of the fiber, where we ultimately aim for the conservation of the cross-section geometry of the preform. In the long term, we aim to develop multimaterial extrusion techniques, which will allow us to integrate multiple materials monolithically in the same print. Alternatively, it is possible to fill the structure with powdered materials such as Si or Ge. The filled structure can be then sintered in order to obtain the preform. Gumennik et al. have described a similar approach [47].

If there is interest in producing a fiber with soda-lime glass as a core material, one simple approach would be to print a suspended-core preform. Suspended-core fibers are of interest for various applications, including sensing [48] and low-loss terahertz guidance [49], and are typically realized with polymers [49]. In this type of fiber geometry, the core is supported by struts that connect it to the outer layer. After drawing, these structures are reduced to dimensions of the order of the wavelength of interest, in a way that, for the guided light, the effect is that of a suspended core. Monro et al. have reported suspended-core fibers in various materials such as silica and bismuth glass [48]. They also provide a series of examples of sensing applications of these fibers, particularly in biological and chemical sensing, confirming viability of these fiber geometries.

Novel approaches to stereolithography-based 3D printing of glass using custom resins have been designed using photocurable silica nanocomposite [50]. Here, we propose a method where glass fiber preforms are fabricated by 3D printing based on off-the-shelf commercial resins, making it a cost-effective and simpler method to achieve 3D-printed glass parts using stereolithography (SLA). The SLA printer (Form 2 by Formlabs) operates using a laser with a wavelength of 405 nm to cross-link the resin through the bottom of the vat as the built platform incrementally rises layer-by-layer.

In order to obtain glass prints, we use a mixture of commercial clear resin (Formlabs FLGPCL04 Clear) with 0.79 mm (1/32") borosilicate glass fibers (#38 Fibre Glast) for the printing process. This allows for great control of the final print material composition and flexibility in achieving the desired properties of the fiber. The glass fibers were dispersed into clear resin using a magnetic stirrer. The glass is added in small increments and homogenized for around 3 min after each addition. For the printing process, the printer was set to open mode to allow for the use of our custom resin. Cuboid preforms of dimensions 4 × 4 × 50 mm were printed at 90° orientation. After printing, the parts were immersed in isopropanol for 10 min to remove excess resin, and post cured with UV light for 30 min at 60 °C. Cured glass preforms with increasing volume ratio of glass fibers mixed into the printing resin (from left to right) are shown in Fig. 2c (I).

The preforms are then post-processed in order to debind the residual resin and sinter the glass particles, resulting in a part composed entirely of glass. The effects of these processes on the preform are illustrated in Fig. 2c (II), where we see a preform after curing (left) and after the baking process (right). In the latter, it is possible to see the black coloration resulting from the baking in an ashing oven, while the tip presents white coloration after debinding. Fig. 2c (III) shows an image taking under microscope of a preform before sintering, where it can be seen that all the resin is indeed baked out of the part, resulting in an interconnected structure formed only by the glass fibers. Several parameters must be controlled, such as baking temperature and heating profile, in order to control chemical reactions and shrinkage. The thermal debinding of the binder was achieved using an ashing oven. The resulting brown parts were sintered in a high-temperature tube oven. Sintering was performed at a temperature of 1300 °C and pressure of 5 × 10^−2^ mbar, following the prescribed protocol in [51]. A holding phase at 800 °C was set to evaporate molecular-bound water and surface-bound silanol groups. Sintering under vacuum enhances the optical transparency since it reduces the trapping of air inside the sintered glass part [52].

After the post processing, the volume and weight of the parts are measured in order to obtain the density. This is then compared to the average density of the print material—composed of the curable resin and glass fiber mixture—which is calculated by *ρ*_avg_ = *ρ*_resin_ (1 − *χ*_v_) + *ρ*_glass_ (*χ*_v_), where *ρ*_resin_ = 1.17 g/cm^3^ is the density of the cured clear resin, *ρ*_glass_ = 2.55 g/cm^3^ is the density of the milled glass fibers, and *χ*_v_ is the volume ratio of the glass fibers mixed into the resin.

The results are shown in Table 2 and graphically in Fig. 2c (IV), where we present the printed parts density for different volume proportions of glass fiber in the resin, compared to the average density of the print material. We observe that, for lower volume ratios of glass mixed into the resin, the obtained results fall within the expected. At higher glass concentrations, however, we see a deviation from the linear behavior of the ideal density. This can be attributed to the sedimentation during the print, which becomes significant at these volume ratios, thus effectively producing higher concentrations of glass in those prints. The printing process happens at the bottom of the tank, where the glass concentration, due to sedimentation is growing constantly during the printing. It begins from (and above) the initial uniform concentration that should fall on the calculated curve.

**Table 2:** Printed parts measurements and expected densities for different volume ratios, as well as measured experimental volume ratios

vol%	Average weight	Density	Ideal density	Corrected
	(g)	(g /cm^3^)	(g/cm^3^)	vol%
0	0.94	1.17 ± 0.01	1.17	0
3.77	0.98	1.22 ± 0.01	1.22	3.62
7.27	1.02	1.28 ± 0.03	1.27	7.97
10.52	1.05	1.31 ± 0.01	1.32	10.14
13.56	1.08	1.35 ± 0.01	1.36	13.04
16.4	1.16	1.45 ± 0.01	1.40	20.29
19.05	1.25	1.56 ± 0.02	1.43	28.26
21.54	1.29	1.61 ± 0.02	1.47	31.88

From Table 2, we can see that at 21.54 vol% milled glass fibers infill parts, the measured density was 1.61 g/cm^3^, which means the actual volume proportion is 31.88 vol%. During the experiment process, we got a sample with actual 34.78 vol% milled glass fibers infill. This result was not far away from the 37.5 vol% silica infill which was fabricated by Kotz et al. [53], where a custom resin was developed, in contrast to the commercial resin we use in this work. Future directions for this work include heat treatment procedures, part sintering, and the study of the mechanical and optical properties of the resulting parts.

### Coherent Material-Selective Capillary Breakup and Segregation Control of Doping

**Fig. 3:**
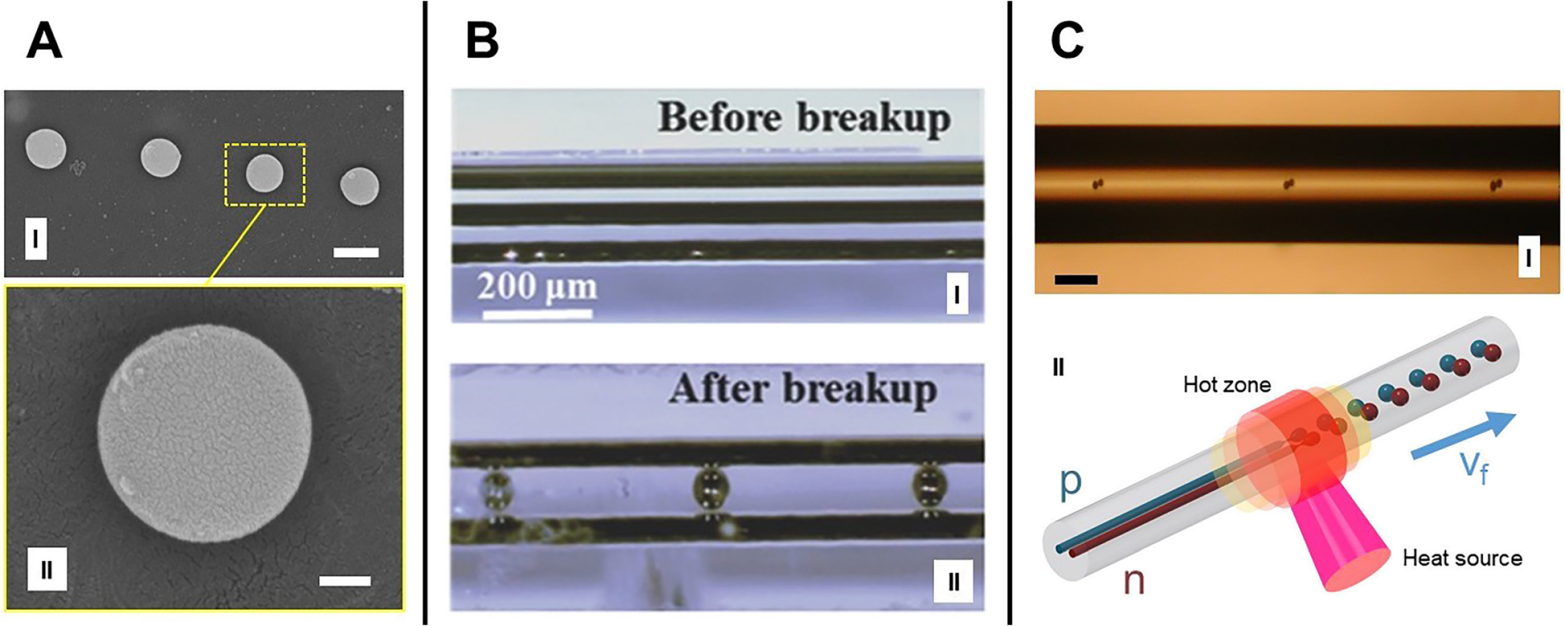
Capillary breakup: **a** (I) Fiber section showing Si spheres formed through capillary breakup (scale bar 0.5 μm). **a** (II) Single sphere image depicting shape quality (scale bar 100 nm), from Gumennik et al. [26]. **b** (I) Example of metal-semiconductor-metal photodetecting device in a single silica fiber before **b** (I) and after **b** (II) breakup process, from Wei et al. [54]. **c** (I) Breakup of a double-core fiber into bi-spherical clusters (scale bar 100 μm), from Gumennik et al. [26]. **c** (II) Schematic representation of the process shown in **c** (I), with p- and n-type cores shown in blue and red, respectively

Once a preform is thermally drawn, the result is a long, thin fiber in which the cross section is preserved, as shown in Fig. 1a (III). In the case of a fiber with one or more cores, these can be axially patterned through a spatially coherent, material-selective capillary breakup process [26], shown in Fig. 3. While the Tomotika model explores the formation of periodic instabilities in an infinite, uniformly heated cylinder of fluid, accounting for the effects of the surrounding fluid [55], propagating Rayleigh instabilities introduces the concept of front propagation [56]. Although several examples of mathematical treatments of capillary instabilities can be found in the literature, such as in Liang et al. [57], these works mostly focus on isothermal regimens, which are not applicable in our case. Moreover, our case has an additional complication of propagating thermal gradient and thus gradient of viscosity. As such, it is probably closer to the marginal instability criterion-driven process, as mentioned in Powers et al. [56]. By combining both our computational and experimental results, we aim to establish a mathematical model for the fiber breakup via axial thermal gradient phenomena.

The present stage of capillary breakup simulation focuses on the stationary regime. We are interested in analyzing how instabilities first develop in an initially stationary fiber subjected to a thermal gradient. This process is analogous to the initial step of the capillary breakup experiments performed by Gumennik et al. [26], where a silica fiber with a 4-μm-thick Si core is fed through the hot zone of a hydrogen-oxygen flame at flows of 0.3 and 0.1 L/min, respectively. Although the maximum temperature achievable with this type of torch can reach up to 2800 °C, the maximum temperature experienced by the fiber must be below the Silica boiling point of 2230 °C.

Considering that the flame width is between 3 and 3.5 mm and that the fiber has a diameter of about 300 μm, it is easy to understand why it is not feasible to experimentally measure the temperature gradient to which the fiber is exposed. Furthermore, the dynamic nature of the breakup process and the fact that it involves multiple materials with varying emissivities further adds to the complexity of the problem, while the fiber’s high aspect ratio and sharp viscosity ratios render the numerical simulation of the full Navier-Stokes equations computationally challenging.

**Fig. 4:**
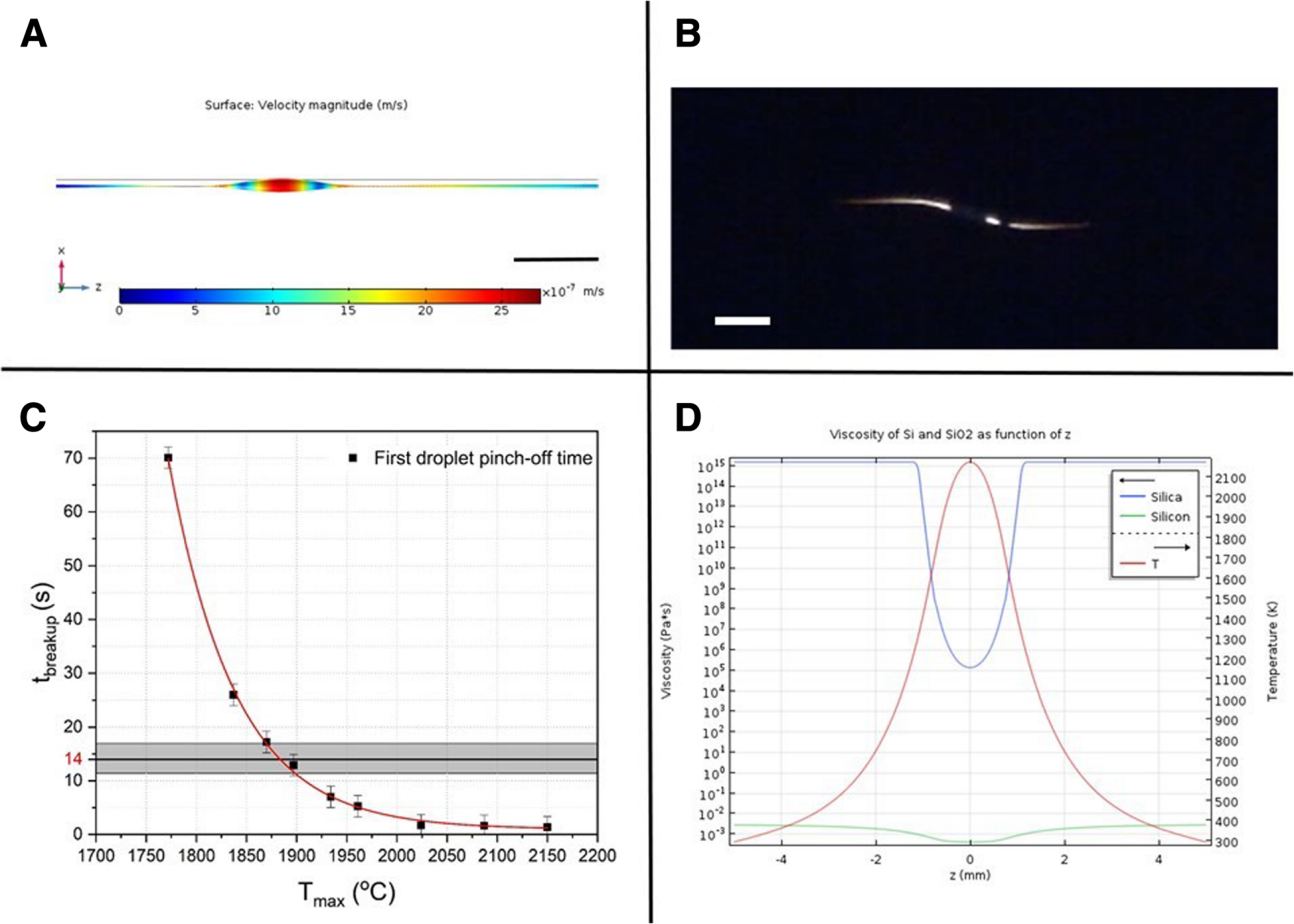
Capillary breakup simulations**: a** Image of the simulated Si core during the first droplet formation, right before the pinch-off for *T*_max_ = 1900 °C. Color scale represents surface velocity (scale bar = 50 μm). **b** Snapshot of fiber breakup experiment recording just after the first droplet pinch-off, indicating a breakup time of 14 ± 3 s (scale bar = 60 μm). **c** Breakup time *t*_breakup_ for different values of *T*_max_. The exponential dependence of *t*_breakup_ on *T*_max_ is evident, a behavior that is expected due to the dependence of *t*_breakup_ on the core’s viscosity, which in turn depends exponentially on the temperature. The shaded rectangle encompasses the temperature range compatible with this breakup time-scale. **d** Si core and SiO_2_ cladding viscosities as a function of axial position, with temperature pro le over-imposed, for *T*_max_ = 1900 °C

In our simulations (using COMSOL Multiphysics® 5.3a, with its Microfluidics and Heat Transfer modules) shown in Fig. 4a, we assume an axisymmetric fiber with radius *r*_fiber_ = 140 μm, composed of a thin Silicon core (*r*_core_ = 2 μm) enclosed in a Silica cladding. Initial heat-transfer simulations showed that a fiber length of 15 mm is sufficient to ensure that the fiber extremities remain at room temperature, thus avoiding influence from the boundaries to the breakup process. Through the sweep of different parameters such as heat source length and distance to the fiber surface, as well as power, we can observe the changes produced in the breakup behavior, thus collecting information about the temperature gradient. Also of importance are the breakup period and speed, parameters which can be compared to experimental results and used for the refinement of the simulations.

The simulations are performed in two steps: first, the steady-state temperature profile is calculated for different heat source powers, in order to achieve the desired maximum temperature *T*_max_. The results are then exported to a time-dependent fluid-flow simulation, where the Navier-Stokes equation is solved with time steps of 0.05 s, from 0 s until the first droplet pinch-off (*t*_breakup_), which is dependent on *T*_max_, as can be seen in Fig. 4c, where *t*_breakup_ is plotted for different values of *T*_max_. It is possible to observe that *t*_breakup_ has an exponential dependence on *T*_max_, which is expected since *t*_breakup_ is proportional to the core’s viscosity, which in turn is exponentially dependent on the temperature.

The relevant parameters used in these simulations are listed in Table 3. Preliminary simulations indicate that the phase transition of the Si core has no significant influence on the steady-state temperature pro le obtained, neither on the subsequent fluid-flow simulations since, in the entire region where the capillary instabilities occur, the temperature is higher than the melting point of Si. Therefore, in order to maintain the model as less computationally intensive, we simulate a core of liquid Si as a first approximation. Moreover, although a fully coupled heat-transfer and fluid-flow simulation is preferable, we consider that the effects of coupling bring second-order corrections to the solution and thus can be neglected at this stage. An image of the simulated core during the first droplet formation, right before the pinch-off, is shown in Fig. 4a, for *T*_max_ = 1900 °C. A plot of the densities of silica and silicon, as a function of the axial position (in the simulation, the z-axis) for this particular simulation, is presented in Fig. 4d, with the temperature profile over-imposed.

**Table 3:** List of parameters used for numerical simulations

Parameter	Expression	Value
Core radius	*r*_core_	2 μm
Fiber radius	*r*_fiber_	140 μm
Fiber length	*h*_fiber_	15 mm
Thermal conductivity of Si	*k*_Si_	149 W/mK
Thermal conductivity of SiO_2_	*k*_SiO2_	1.3 W/mK
Si–SiO_2_ surface tension coefficient	*σ*_0_	1.5 N/m
Air–SiO_2_ surface tension coefficient	*σ*_1_	0.75 N/m
Si tangent coefficient of isothermal expansion	*α*_Si_	2.5 × 0^−6^ K ^− 1^
Softening temperature of SiO_2_	*T*_s_	1710 °C
Melting temperature of Si	*T*_m_	1414 °C

Our results allow us to define the ranges of viscosities for which the time scales for breakup are comparable to those observed experimentally, thus assessing qualitatively the temperature profile imposed on the fiber. Fig. 4b shows a snapshot of a fiber breakup experiment recording just after the first droplet pinch-off, at the frame corresponding to *t* = 39 s. Since the resolution of the images before this frame is not ideal, judging on the basis of the symmetry of the breakup behavior, it is possible to assess that the pinch-off of the first droplet occurs at *t* = 35 ± 3 s, which translates to a breakup time after the temperature steady-state regime is achieved, of 14 ± 3 s. The shaded rectangle in Fig. 4c encompasses the temperature range compatible with this breakup time-scale, from which we can infer that the maximum temperature to which the fiber is exposed is 1885 ± 15 °C. With the collection of statistics on the breakup behavior in the future, we aim to develop a procedure for the temperature measurement of the process, which is otherwise unattainable by conventional means [26, 58, 59].

**Fig. 5:**
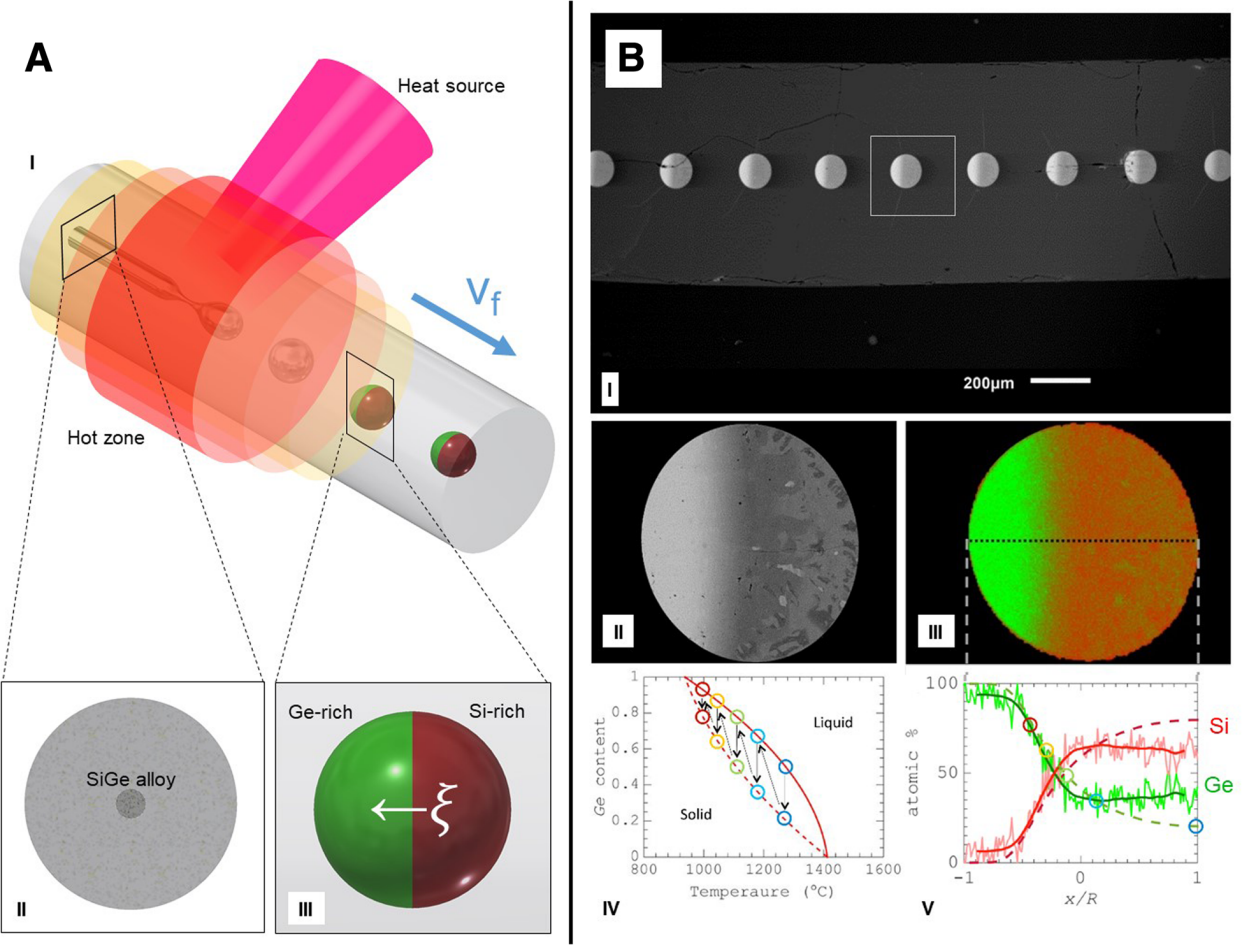
Segregation control of doping: **a** (I) Schematic illustration of the segregation-driven control of doping in post-breakup semiconducting particles. Details: cross section of a Ge-rich Silicon continuous core in silica fiber **a** (II) and post-breakup schematic drawing of doping-segregated sphere, or Janus particle, with the Ge-rich side indicated in green **a** (III). **b** (I) Scanning Electron Microscope image in backscattered mode of the fiber, polished along its axis, showing an array of Janus particles. **b** (II) Detail of single Janus particle cross section. **b** (III) Energy-dispersive X-ray spectroscopy (EDS) map of Janus particle, indicating non-homogeneous distribution of Ge along the cross section, from overlaid maps of Si (in red) and Ge (in green). **b** (IV) Si-Ge equilibrium phase diagram, liquidus (solid line), and solidus (dashed line). **b** (V) SiGe atomic content distribution along the dashed line in **b** (III). (From Gumennik et al. [47])

Moreover, segregation-driven control of doping in post-breakup semiconducting particles is attainable, allowing to control an individual device’s internal architecture. It is possible to control the structure of a single sphere, as was demonstrated by Gumennik et al. [47] shown in Fig. 5. When the droplet is exiting the flame, it experiences a thermal gradient: it is colder on the end distant from the flame, and consequently, it is expected to solidify laterally, starting from the colder side. If the sphere is doped with a material that is more soluble in a liquid than in a solid, this dopant will be repelled into the liquid as the solidi cation front propagates, collecting predominantly on the hot side. This effect is shown schematically in Fig. 1c and can be used to synthesize structured particles, composed for example of Ge-rich Si, (as shown in Fig. 5): Starting with 50:50 Si-Ge mixture in the fiber core, after the breakup the sphere will solidify, such that the Ge is extruded into the melt as the solidi cation evolves, leading to anisotropic distribution and resulting in axially oriented Janus particle heterojunctions [47]. This method can be extremely useful in assembling complex fiber-embedded devices such as heterojunctions, as shown in Fig. 1d and e.

### Biomedical Application

Fiber technology is frequently utilized in various biomedical applications as chemical, biological, and physical sensors. Fiber-embedded sensors have been designed to monitor physical parameters such as stresses, temperature, pressure, and humidity or chemical parameters such as pH level, oxygen concentrations, and carbon dioxide concentrations [60]. Fiber bundles are beneficial to embed multiple sensors together in a single system and in increasing signal reception levels, resulting in higher signal-to-noise ratios. Lightness, flexibility, and unique optical properties are the main characteristics that lead the demand for fiber sensors in biomedical studies. To meet clinical usage requirements, preforms must be fabricated from biocompatible, non-toxic, and chemically inert materials to prevent immune reaction from the patient. Examples of smart fiber development include a neural fiber probe composed of a polymer and metal core composition that enables flexibility and bending stiffness of the neural probe as it provides in vivo optogenetic stimulation and delivers drugs as an input in order to record feedback electrical and physiological output signals [33]. Another example is a fiber integrating microfluidic principles with complex cross-sectional geometries and meter-long microchannels which analyzes cell separation by dielectrophoresis (DEP). Live and dead cells are separated by inertial and dielectrophoretic forces by sheathless, high-throughput microfluidic cell separator which contains conductive materials in the microchannels [14]. The following strategies show a new array of possibilities where smart fibers can be used in biological interfacing.

**Fig. 6:**
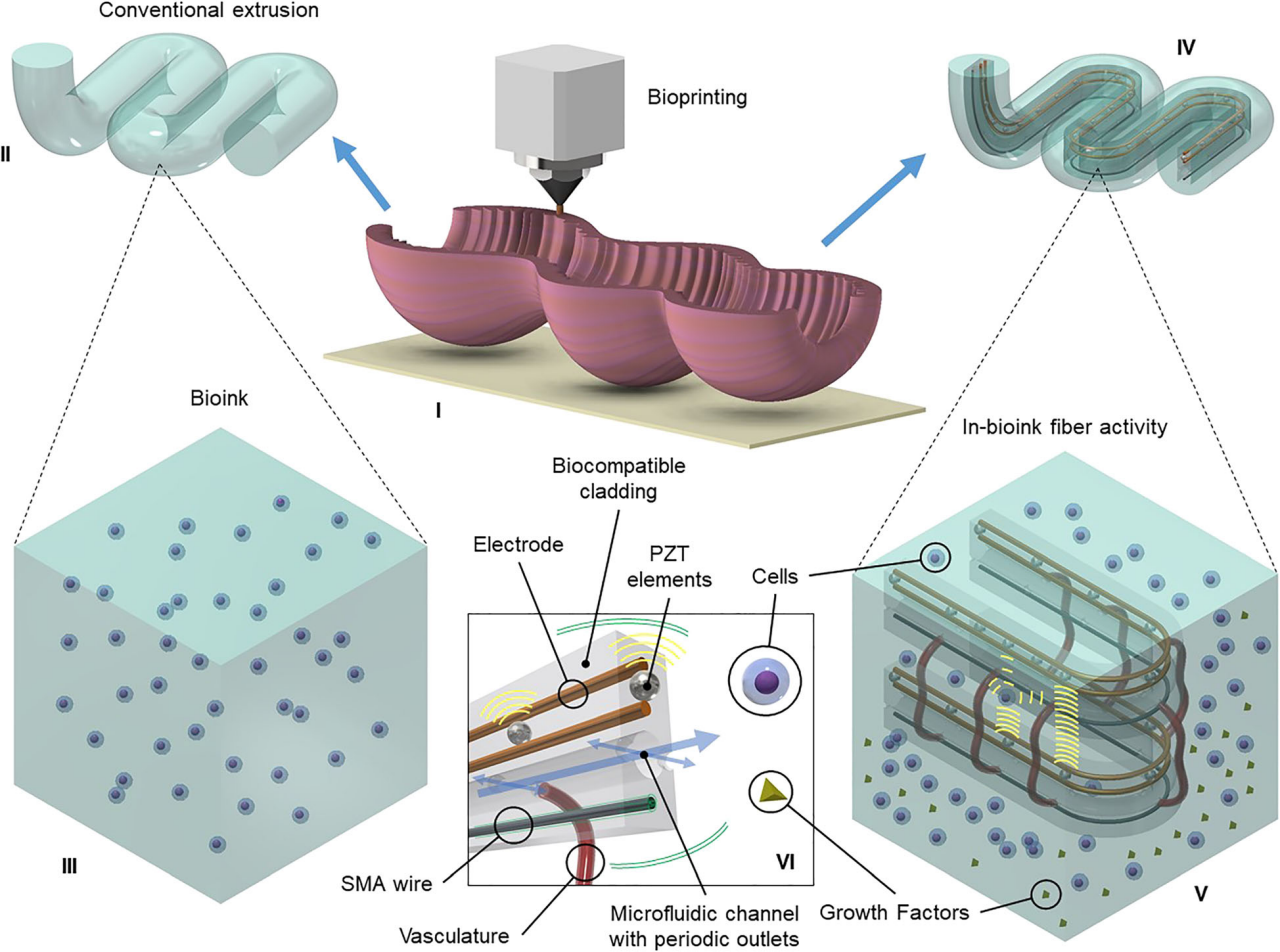
Biological interfacing. (I) Three-dimensional bioprinting of a tissue. (II) Standard microextrusion of bioink. (III) Conventional bioink with cells suspended in hydrogel. (IV) Novel coaxial microextrusion of biointerfacing fiber coated in bioink. (V) Close-up view of fiber where biointerfacing occurs: epithelial cells and vascular epithelial growth factors are excreted from different microchannels and result in cellular self-assembled vasculature between two orifices; piezoelectric elements measure surrounding cell density by ultrasound; and shape memory alloy wires provide peristaltic motion in the tissue. (VI) Visualization of biointerfacing fiber and its components

Consider an artificial gut that can serve as a bioactivity testing platform at the microscale and at the macroscale. With today’s progress in tissue engineering, a variety of functionalities can be integrated in bioink-coated fibers co-extruded using a bioprinter for tissue fabrication, as shown in Fig. 6(I). Traditionally, bioprinting research aims at creating tissue grafts for regenerative medical practice and does so by carefully designing the hydrogel (Fig. 6(II)) with the appropriate nutrition and signaling molecules for the type of cells required based on the application (Fig. 6(III)). Tissue engineering is very challenging to study as the whole biology of the system completely changes microseconds after the experiment has been launched. Monitoring and regularly tuning a tissue’s maturation remains very complex.

We propose here a solution by introducing smart fibers in the design (Fig. 6(IV)) to provide a better understanding of the climate and environmental growth. The embedded fiber holds multiple functionalities (Fig. 6(VI)) such as inducing vasculogenesis, ultrasonic imaging, peristaltic movement, and microfluidic flow. Control of the microenvironment takes place via the fiber hooked to syringe pumps and wired to an analytical software. The features of this application (Fig. 6(V)), including pilot experimental data, are detailed in the next subsections.

### The Extracellular Matrix and Vasculature

Tissue engineering is widely explored with the increase of artificial tissue needs [61, 62], and the ability to bioprint realistic tissue has an important role to play in tomorrow’s drug and treatment development [63, 64]. One of the biggest challenges is the design of the extracellular matrix (ECM), composed of proteins, growth factors, and other biomolecules, that guide the cell’s contribution to the tissue [65]. Naturally, the ECM gives purpose and structure to the cells, and its extraction typically works by decellularizing tissue and recycling or reusing the ECM for a new cellular construct. The ECM comes in the form of solvents, hydrogels, biopolymers, bioceramics, aerogels, or foams to provide biodegradable or resorbable structure to the tissue [66]. Due to tissue engineering’s high complexity in defining the specifics of the biosystem—mechanical properties, scaffold dissolvability or absorption rates, initial cell types, nutrition, density and ratios, growth factors introduction, and its resulting bioactivity and tissue self-assembly—it is vital to assess the behavior of different types of naturally produced ECM or artificially developed biomaterials in the presence of interacting cells. Moreover, viable tissue requires an organized vascular system that supplies nutrition and oxygen to the tissue for the health and growth of cells. Vascularization provides the natural microfluidic feed of biochemicals to initiate proliferation, specialization, interactions, and motion. The vascular network is formed by vasculogenesis, arteriogenesis, and angiogenesis. Vasculogenesis develops its network through the differentiation and division of endothelial stem cell [67]. Angiogenesis forms new sprouts from existing vessels that are formed in the early embryonic vasculogenesis stage [68].

### In-Fiber Microfluidic Feed

Microfluidic conduits with periodic microchannels for content delivery can be used to weave microfluids to specific locations in tissue constructs. The liquefaction front at the boundary of the hot zone defines the droplets’ pinch-off location as described previously. Multiple cores can therefore be broken up in a spatially coherent manner. For example, a silica fiber including a platinum and a silicon core can become a fiber tube with multiple outlets, by inducing the silicon core into an array of spheres and then thinning the fiber using hydrogen fluoride, etching the silicon spheres with potassium hydroxide and etching the platinum core with regal water. An example of the result is shown in Fig. 6(V). The flexibility of fibers allows the microfluidic feed to be integrated in multiple ways in tissue construct. In Fig. 6(IV), the microfluidic channels are used to provide the necessary cell type and growth factors to initiate vascularization and angiogenesis as the tissue reaches maturity.

### Biomaterial and Biochemical Testing

**Fig. 7:**
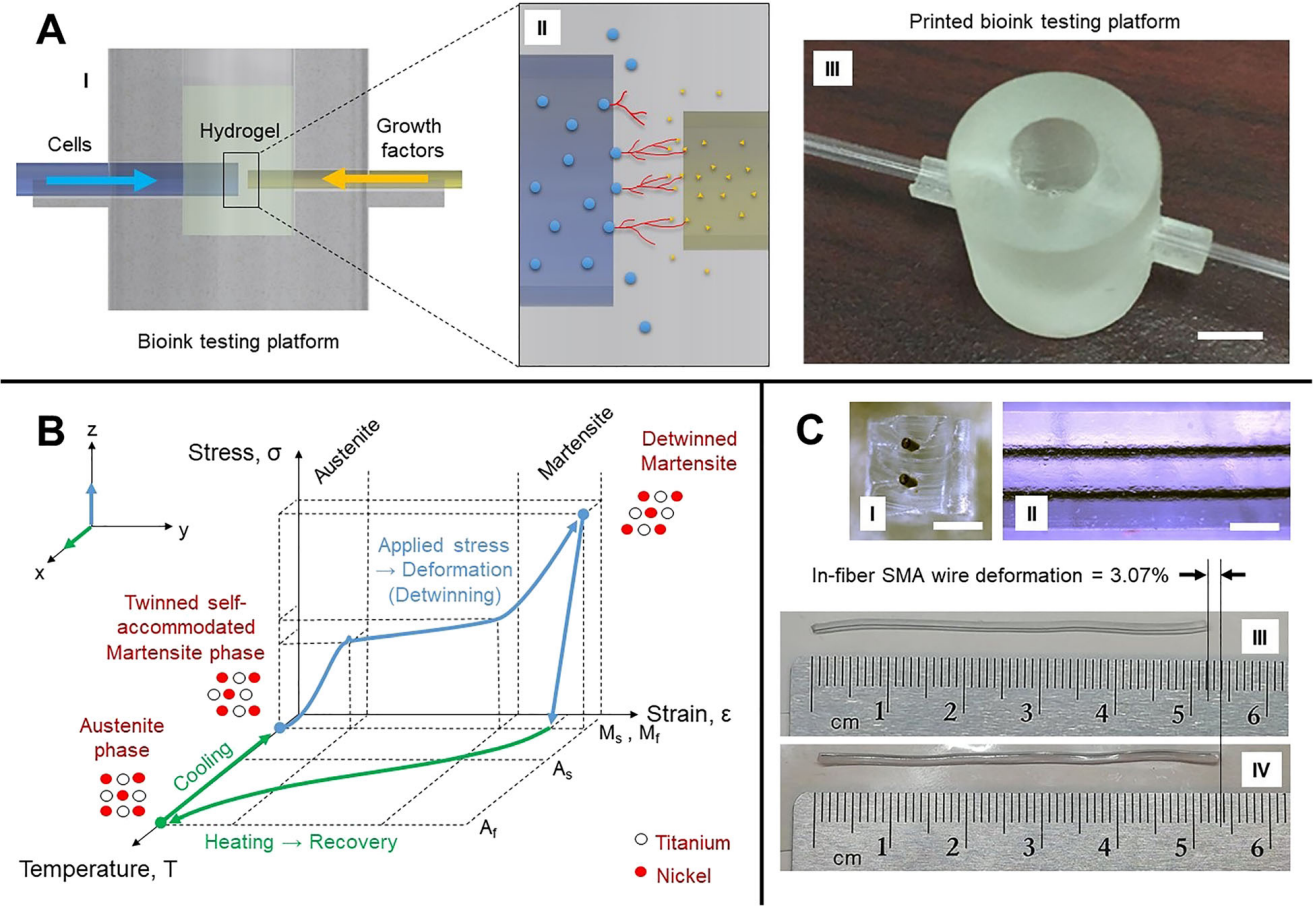
Pilot experiments for biointerfacing: **a** (I) Cross section of biotesting well showing fluidic feed. **a** (II) Example of the interaction of epithelial cells (blue circles on the left) with vascular endothelial growth factors (yellow dots on the right) resulting in vasculogenesis excreted by the cells (red lines in the center). **a** (III) Printed testing platform in biocompatible resin (scale bar = 5 mm). **b** Temperature-stress-strain graph of the shape memory effect. **c** (I) Cross section of in-fiber SMA wires (scale bar = 1 mm). **c** (II) Side view of in-fiber SMA wires (scale bar = 1 mm). **c** (III) 5.2-mm segment of an in-fiber SMA wire in contraction after being heated at 80 °C. **c** (IV) 5.36-mm segment of extended in-fiber SMA wire at room temperature

In parallel to fiber development, a new testing platform (Fig. 7a (I)) was designed to analyze vascularization and cell-to-cell interactions in the presence of growth factors (Fig. 7a (II)). The platforms were printed in high resolution at an orientation of 30 from biocompatible resin by stereolithography (Formlabs’ Form 2 and Dental LT Clear resin). The print result, shown in Fig. 7a (III), was assembled with two glass capillary tubes with outer diameters of 1.8 mm and 1.0 mm where biological agents are fed. Initial trials will assess diffusion parameters of biochemicals and the growth of cellular colonies in various biocompatible materials. These platforms are designed to be single-use. In 2 h, 24 testing wells can be printed at a relatively low cost. The distance between each capillary outlet of two parallel fibers can be adjusted between 100 and 400 m to investigate the optimum vasculogenesis range that is accepted around 200 m [67]. The medium of interaction in the well’s chamber will first host commercial bioinks (Cellink, USA), one containing sodium alginate and nanofibrillar cellulose and the other containing gelatin methacryloyl, before developing our own in-house biomaterials. The glass capillary tubes shown in Fig. 7a (I) will eventually be replaced by the microfluidic fiber shown in Fig. 6a (V) which will be discussed in the next section.

### Peristaltic Motion

Shape memory alloy (SMA) wires are lightweight, non-corrosive, and cost-efficient actuating materials for refined applications in a variety of applications such as prosthetic biomimicry [69], self-expandable surgical implants [70], and aerospace engineering [71].

SMAs are metal compounds known for their shape memory effect and pseudoelasticity. Although such properties are typically found in nickel-titanium, these properties can be found in a range of different other metal alloys. Figure 7(b) shows the shape memory effect in terms of temperature, stress, and strain. As shown, at low temperatures, the SMA in its martensite solid state can be deformed by mechanical force, and when thermally induced, goes through a non-diffusive molecular reordering, converting to an austenite solid state. When cooled, the material will return to its initial martensite form, hence the shape memory effect. This thermal cycle is defined by four temperatures, the starting and finishing martensite and austenite temperatures (*M*_s,_ *M*_f_, *A*_s_, and *A*_f_), which specifies the start and end of transition periods between states. When the SMA is deformed in as martensite, the molecular de-ordering is defined as detwinning, and it allows the material to experience elongation, which is particularly useful for actuation applications. Essentially, the shape memory effect cycle can occur hundreds of times for an average elongation of 6% and contraction [72], hence its nickname “muscle wire” for its close similarity to muscular myofibrils.

This unique characteristic was first reported by Alden Greninger and Victor Mooradian in 1938 [73], can also be triggered by magnetic field energy [74], namely ferromagnetic shape memory alloys (FSMA), and can be found in polymers (SMP) as well [75]. Today, SMA’s mechanical fatigue and fracture, elasticity, and thermodynamics have been characterized well experimentally [76] and mathematically [77], and its behavior has been modeled [78].

Although the shape memory effect allows for nice contraction behavior of a material, for appropriate robotic applications, the motion needs to be reversable. Typically, an SMA is set in tandem with an opposite mechanism, such as springs, electric drives, elastic bands, or simply another SMA wire. Furthermore, the assembly changes whether it is a linear or rotatory actuation and if the opposing contractions of the actuation are equal. Although wires are thin and weak alone, they can be bundled together to reach the desired force and keep its shape memory effect response time. SMA wires can also be coiled around a capstan to provide greater elongation over shorter distances. Various strategies have been reviewed and chosen for specific applications [79]. The thermal induction is typically best controlled by powering the SMA wire and varying the input current of the order of hundreds of milliamperes. Cooling can be done naturally or by including heat sinks and ventilation.

Fiber drawing technology allows us to play on different material characteristics and to provide to an SMA wire an elastic coating that helps preserve the disorder state of the SMA fiber. As shown in Fig. 7c (I) and c (II), a fiber can be drawn with multiple embedded SMA wires. Styrene-ethylene-butylene-styrene (SEBS) was chosen as the surrounding structure to the SMA wires. SEBS is a copolymer elastomer that can withstand the drawing temperature of 80 °C. While preliminary results show that the actuation can work but with lower efficiency than bare SMA wires, the back-and-forth motion through heating (Fig. 7c (III)) and cooling (Fig. 7c (IV)) cycles is observed for a deformation of approximately 3.07%. More research is required to optimize the setup, but this early stage of experimentation in-fiber SMA actuation shows promising outlooks. The wires were physically pulled and heated using a hot plate to prove the concept. Heating by current would allow speeding up the shape memory effect and controlling better the heat’s diffusion through the fiber. The 5.35-mm wire was measured to have a diameter of 0.11 mm and a resistance of 18.2 was recorded across the fiber segment. To provide a frame of reference, according to SMA wire manufacturers (Dynalloy, Inc.), a 0.1-mm-thick wire made of nickel and titanium requires approximately 200 mA of current for a 1 s contraction. Controlling each wire individually would allow for directional movement, and adding more SMA wires to the design would allow multi-directional motion and greater contraction strength. Moreover, if ferromagnetic SMA wires were used, the control of the motion could become locally controllable via tuning of the interacting magnetic field. Additionally, the FAMES Lab’s drawing tower having the ability to rotate a preform as it is drawing into a fiber enables the possibility to manufacture spring-like structures of SMA wires in the fiber which allows for greater deformation, similar to commercially available SMA springs. Clearly, varieties of strategies are enabled with in-fiber SMA wires.

### Biosensing

Biosensors are developed in a wide variety of ways. They can be designed label-based or label-free to detect specific expressions from biological elements such as cells, bacteria, hormones, proteins, DNA, and more [80, 81], from sampling blood, urine, saliva, sweat, or tears. Psychophysiological conditions can be observed from real-time biofeedback such as blood pressure, electrodermal activity, skin conductance, respiration and heart rates, and more [82, 83]. Bioimaging has been done by optical imaging, ultrasound, magnetic resonance frequency, computed tomography, near-infrared spectroscopy, quantum dot probing, and by many more techniques [84].

In optical fiber research, previous biosensing fibers have been fabricated relying on silicon photonic crystal detection of biological radiation [85]. Photonic crystal technology has been used before to monitor in label-free real-time cellular morphology and survival [86]. Such progress in biophotonics has led to hollow-core microstructured fibers visible under magnetic resonance imaging (MRI) [87]. The hollow core allows the propagation of the optical radiations along the fiber over very long distances. The geometry of the hollow fibers varies to tune the photonic bandgaps and dispersion of different detected wavelengths. The inner walls of these hollow tubes are coated with oppositely charged polyelectrolytes and magnetite nanoparticles which are used as contrast agents for MRI. Such design therefore enables new biomedical precision diagnosis opportunities, for example, in the observations of neural activity in vivo [87, 88].

Ultrasonography technology enables us to observe density of cells in liquid or gel in a non-intrusive manner [89, 90]. Ultrasonic probes typically function according to the piezoelectric effect (generation of electricity from applied stress), which was first discovered by Jacques and Pierre Curie in 1880 [91]. The inverse piezoelectric effect (deformation of a piezoelectric crystal from an applied electric field) was induced mathematically by Gabriel Lippmann in 1881 [92] and later in 1916, Chilowsky Constantin and Paul Langevin developed ultrasonic submarine detection for World War I military applications [93]. Thereon, sonar applications have been diverse, such as underwater imaging and fish-finding [94, 95] and energy harvesting [96, 97].

The piezoelectric elements convert electrical energy to and from mechanical energy and transmit sound waves between each other. All frequency and bandwidth parameters require precise regulation, and good energy transmission requires good acoustic and damping matching impedances. Two fibers with integrated piezoelectric elements, designed as pulsing emitter and receiver, can create an ultrasonic waveguide between each other to measure density over the wave’s trajectory. This fiber enables us to sense the microstructures of the environment as the tissue reaches maturation. The piezoelectric elements are created by capillary breakup from a PZT core for example. PZT elements have an acoustic impedance of 33.7 × 10^6^ kg/m^2^s with a resonance frequency below 25 MHz. The produced spheres are lined with conductive electrodes to a transducer. This setup provides the feedback in a control system to better adjust microfluidic and motion feed. The in-fiber ultrasonic imaging of the microenvironment clearly helps understand how the tissue environment behaves over time.

## Conclusions

We have formulated the concept of VLSI for fibers (VLSI-Fi)—a combination of liquid-phase processing techniques in microelectronic materials forming a toolbox for fabrication of high-performance devices and systems in fibers and textiles. Our experimental work focuses on a set of demonstrations substantiating our control over narrower aspects of VLSI-Fi, such as preform 3D printing, in-fiber circuit assembly by material-selective spatially coherent capillary instability, and segregation-driven doping control at the level of an individual fiber-embedded device. We envision that VLSI-Fi will enable realization of product in multiple technological areas, one of which is fabrication of active biomimetic scaffolds for engineered tissues with realistic microstructures.

## Data Availability

Not applicable.

## Abbreviations

BJT: Bipolar junction transistor
CAD: Computer-aided design
CMOS: Complementary metal-oxide-semiconductor
DEP: Dielectrophoresis
ECM: Extracellular matrix
EDS: Energy-dispersive X-ray spectroscopy
FAMES Lab: Fibers and Additive Manufacturing Enabled Systems Laboratory
FOS: Fiber optic sensors
FSMA: Ferromagnetic shape memory alloys
HPCVD: High-pressure chemical vapor deposition
IoT: Internet of Things
MOF: Microstructured optical fibers
MOSFET: Metal-oxide-semiconductor field-effect transistor
MRI: Magnetic resonance imaging
PCF: Photonic crystal fiber
PZT: Lead zirconate titanate
SEBS: Styrene-ethylene-butylene-styrene
SLA: Stereolithography
SMA: Shape memory alloy
SMP: Shape memory polymers
TPC: Trans-Pacific undersea cable
UV: Ultraviolet
VLSI: Very large-scale integration
VLSI-Fi: Very large-scale integration for fibers
